# Delayed Activation of T Cells at the Site of Infection Facilitates the Establishment of Trypanosoma cruzi in Both Naive and Immune Hosts

**DOI:** 10.1128/msphere.00601-22

**Published:** 2023-01-25

**Authors:** Angel M. Padilla, Charles Rosenberg, Peter Cook, Fernando Sanchez-Valdez, Caroline McElhannon, Rick L. Tarleton

**Affiliations:** a Department of Cellular Biology, University of Georgia, Athens, Georgia, USA; b Center for Tropical and Emerging Global Diseases, University of Georgia, Athens, Georgia, USA; c Department of Microbiology, University of Georgia, Athens, Georgia, USA; d Medical Research Council Centre for Medical Mycology, University of Exeter, Exeter, United Kingdom; University at Buffalo

**Keywords:** T cells, *Trypanosoma cruzi*, site of infection

## Abstract

Although parasite entry through breaks in the skin or mucosa is one of the main routes of natural transmission of Trypanosoma cruzi, little is known about the host cell types initially invaded nor the ability of those host cells to initiate immune responses at the site of infection. To gain insights into these early events, we studied the fate of fluorescently tagged T. cruzi delivered subcutaneously in mouse footpads or ears. We demonstrate that the majority of parasites introduced into the skin initially proliferate there until 8 to 10 days postinfection, when the parasite load decreases. This decline in parasite numbers is dependent on the presence of an intact T cell compartment and on the ability of hosts to produce gamma interferon (IFN-γ). Many of the parasite-containing cells at the initial infection site display a macrophage/monocyte phenotype but with low expression of activation markers, suggesting that these cells provide an early niche for T. cruzi proliferation, rather than being active in parasite control. It is only after the first round of T. cruzi replication and release from host cells that signs of immune activation and control of parasites become apparent. The delay in the activation and failure to rapidly control parasite replication are observed even when T. cruzi-primed T cells are present, such as in chronically infected mice. This failure of a primed immune system to recognize and react prior to extensive parasite expansion at the infection site likely poses a significant challenge for the development of vaccines aiming to prevent T. cruzi infection.

**IMPORTANCE** Trypanosoma cruzi, the parasite causing Chagas disease, usually infects through the mucosa or breaks in the skin, but little is known about the parasite's fate at the site of entry or the early events involving immune control there. Here, we track the local proliferation and subsequent dissemination of fluorescently tagged T. cruzi and the initial immune response at the point of entry. We show that T. cruzi preferentially infects innate immune cells in the skin and that the stimulation of an adaptive T cell response does not occur until after the release of parasites from this first round of infected host cells. This first immunologically “silent” proliferation occurs even in the presence of a strong immune T cell memory generated by previous infection. This capacity of T. cruzi to establish infections while avoiding initial immune recognition has important implications for the potential to develop vaccines to prevent T. cruzi infection.

## INTRODUCTION

Millions of people are exposed to natural Trypanosoma cruzi infection, particularly in rural areas of Central and South America; to date, no effective vaccine to prevent infection in humans or to block transmission in animal reservoirs has been developed. Understanding the immune mechanisms in the very early stages of infection could provide insights into how T. cruzi establishes and then maintains a persistent infection. Such knowledge could be invaluable for determining the requirements for and the chances of developing an effective vaccine.

Natural infection by T. cruzi frequently occurs through the mucosa or when breaks in the skin come in contact with parasite-containing feces deposited by the insect vector. T. cruzi infection elicits very potent adaptive immune responses that generally allow hosts to tightly control the parasite burden, resulting in relatively few acute-infection-related deaths. However, despite the strength and effectiveness of these adaptive immune responses, they are relatively slow to develop, being detectable only ~8 to 10 days postinfection (1). The relative lack of Toll-like receptor (TLR) ligands displayed by T. cruzi has been suggested to contribute to this slow development of adaptive responses (2, 3). However, the influence of the cells infected by T. cruzi at the site of entry and their role in initiating the antiparasite immune response have not been fully addressed. Furthermore, there are a number of contradictory reports concerning the activation status of *T cruzi*-infected cells capable of antigen presentation, with some *in vitro* studies suggesting that T. cruzi-infected antigen-presenting cells (APCs) are highly activated, while other studies suggest a specific impairment of these cells due to active interference by the parasite (4–7). Even less is known concerning related immune evasion mechanisms in play during infection in the skin. These early events at the site of entry may influence the timing of the development of the immune response and thus impact the kinetics of parasite proliferation and dispersion in the host.

Studies on the early stages of T. cruzi infection have usually been limited due to a lack of tools to follow both parasite development and the elicited antiparasite immune response *in vivo*. Here, we describe a model of infection using bioluminescent and fluorescently tagged T. cruzi parasites, which allows us to track parasite development *in vivo* and to identify the infected cells at the site of parasite entry soon after infection. This approach revealed an underappreciated aspect of T. cruzi infection, i.e., parasites initially invade and proliferate prior to triggering a controlling immune response, and emphasizes the role of gamma interferon (IFN-γ)-producing CD4^+^ T cells in the control of the parasite load at the site of entry.

## RESULTS

### T. cruzi infects and proliferates predominantly at the site of initial parasite entry.

**FIG 1:**
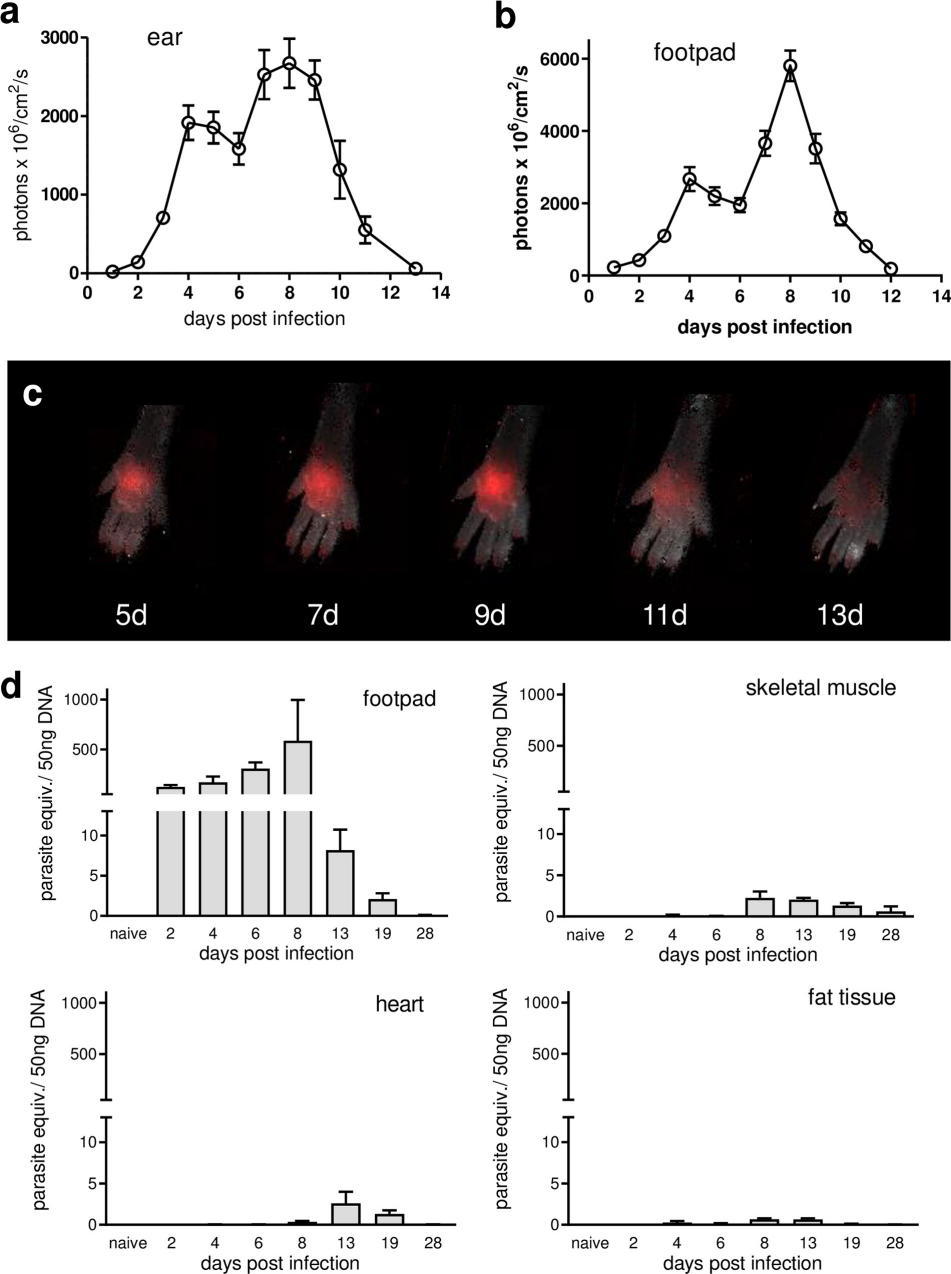
T. cruzi proliferates at the site of parasite entry. (a and b) C57BL/6 mice were infected in the ears (a) or footpads (b) with 2.5 × 10^5^ CL-tdTomato-expressing trypomastigotes, and the parasite load at the site of the infection was determined by *in vivo* imaging. (c) Representative composite images of the footpads, depicting the kinetics of fluorescence intensity through the initial days of infection, are shown. (d) Parasite levels at the site of infection and in skeletal muscle, heart, and fat tissue were determined by rtPCR at different times postinfection.

In order to mimic the natural route of infection through the skin, we infected C57BL/6 mice subcutaneously in the footpads or the ears using T. cruzi parasites expressing the fluorescent protein tdTomato (8), which allowed us to track the kinetics of parasite proliferation *in vivo* during the early infection period. As expected, fluorescent signals at the site of infection increased over time, indicating invasion of and replication within host cells near the infection site. Fluorescence intensity reached a peak approximately 8 days postinfection and then sharply decreased to levels below the limit of detection by this method between 10 and 15 days postinfection (Fig. 1a to c). Parasite numbers at the site of infection also decreased 4 to 6 days postinfection in both the ear and the footpad, coincident with the expected first round of release of trypomastigotes from infected cells and their reinvasion of other host cells locally and presumably at sites distant from the initial infection site. This scenario was supported by the use of noninvasive *in vivo* imaging at a resolution that allowed us to observe individual parasites at the site of infection. After injection in the ear, free motile fluorescent trypomastigotes were readily detected at the site of infection (see Video S1 in the supplemental material). From 24 h to approximately 4 days postinfection, no free motile parasites were visible at the site, suggesting that the parasites either had invaded host cells and converted to nonmotile amastigotes or were no longer motile/viable (see Video S2). In some cases, the apparent movement of intracellular trypomastigotes within soon-to-be-disrupted host cells could be observed at 4 days postinfection (see Video S3) and motile extracellular parasites were again detected at the infection site at 5 days postinfection, suggesting completion of the initial replication cycle inside the host cells (see Video S4).

Real-time PCR (rtPCR) measurements of parasite loads also confirmed these kinetics of parasite replication at the infection site in the footpads and documented the delayed dispersion of T. cruzi outside the initial site of infection, into skeletal muscle, heart, and fat, all of which are known target tissues for infection and chronic persistence of T. cruzi (Fig. 1d).

Collectively, these results indicate that T. cruzi predominantly invades and replicates at the site of entry during the first week after skin infection. The cycle of host cell infection and release *in vivo* is similar to that described previously *in vitro*, with 4 to 5 days being required for completion and the emergence of a much expanded (presumably ~500-fold) population of parasites capable of reinvasion and dissemination to new sites (9). At least two questions arise from these observations. What cell types are the primary host cells for proliferating parasites at the site of entry? What mechanisms are responsible for the abrupt decrease in parasite load at the site of entry beginning at day 8 to 9 of infection?

### Macrophages and monocytes are the main leukocyte populations containing parasites at the site of infection.

**FIG 2:**
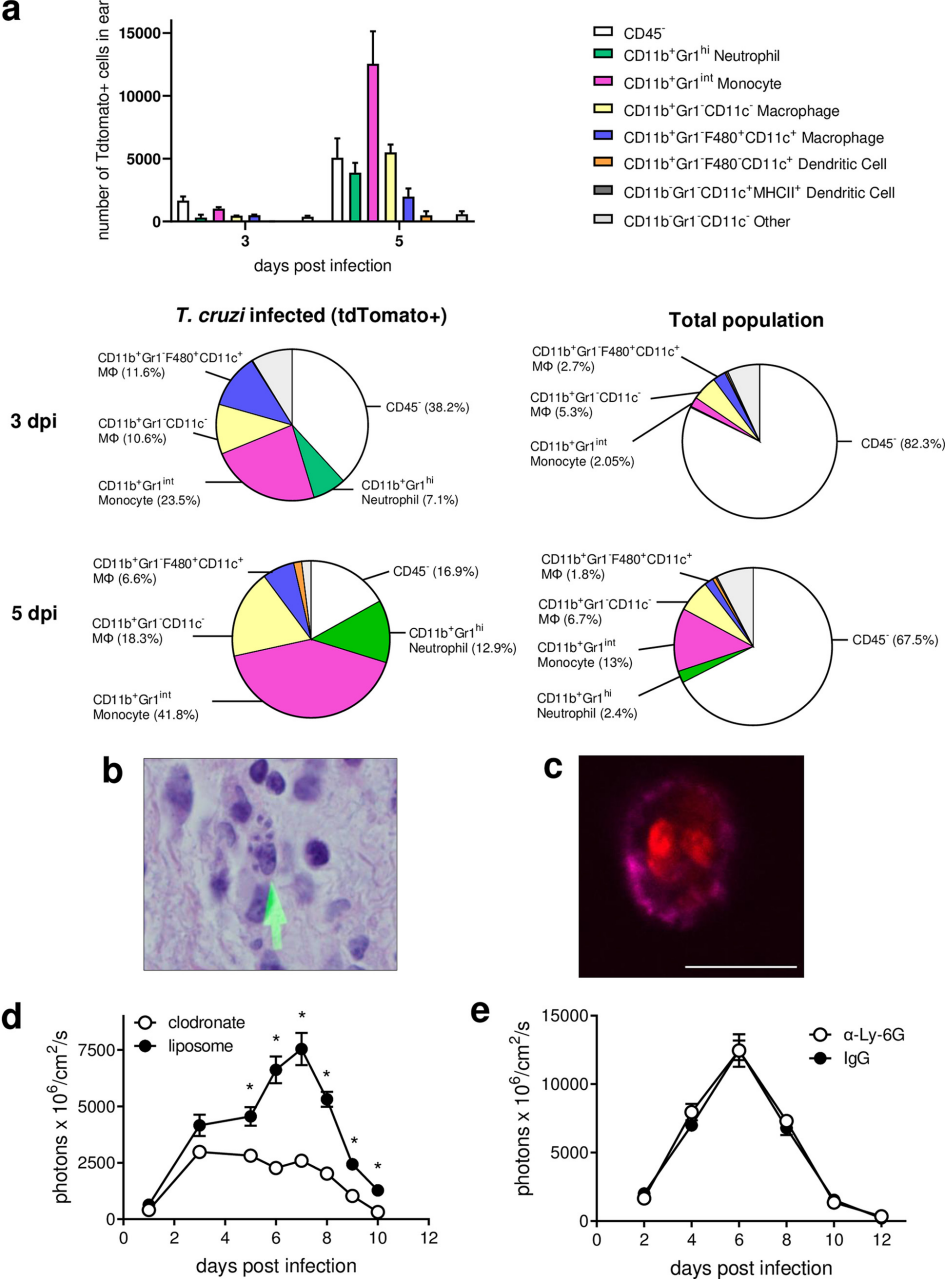
Macrophages and monocytes are the main leukocyte populations containing parasites at the site of initial infection. (a) Cells from ears of C57BL/6 mice infected with CL-tdTomato-expressing parasites were isolated by enzymatic digestion, and the different cell populations containing parasites were assessed by flow cytometry at 3 and 5 days postinfection. Pie graphs represent the proportions of cell populations with respect to the total tdTomato-containing cells (tdTomato+) (left graphs) and the proportions of each cell population with respect to the overall cell composition at the site (total population) (right graphs). (b and c) The presence of infected macrophages (b) and CD11b^+^ cells (c) at the infection site in the ears was confirmed by histological sectioning and fluorescence microscopy of cells released by enzymatic digestion, respectively. The arrow in panel b indicates a macrophage harboring T. cruzi amastigotes. tdTomato-expressing intracellular amastigotes (red) inside a CD11b^+^ cell (magenta) isolated from the infected ears were identified by fluorescence microscopy (c). Scale bar, 10 μm. (d and e) The effects of the depletion of macrophages (d) and neutrophils (e) by administration of clodronate-containing liposomes and anti-Ly-6G antibody, respectively, in C57BL/6 mice infected with tdTomato-expressing T. cruzi were determined by *in vivo* imaging of the infected feet. *, *P* < 0.05 (Mann-Whitney test). Mϕ, macrophage.

To address the first question, we isolated cells from the infected ears and identified those containing parasites based on the fluorescence of the tdTomato-expressing T. cruzi. At 3 days postinfection, approximately one-half of the parasite-containing cells isolated from the ear were CD45^+^ leukocytes; by 5 days postinfection, CD45^+^ cells constituted the majority of the infected cells recovered at the site of entry (Fig. 2a). Macrophages (CD11b^+^ Gr1^−^ CD11c^−^ and CD11b^+^ Gr1^−^ F4/80^+^ CD11c^+^), monocytes (CD11b^+^ Gr1^int^), and neutrophils (CD11b^+^ Gr1^high^) were the predominant leukocyte populations containing parasites at these early time points after infection, with macrophage/monocyte populations clearly dominating by 5 days postinfection. Relatively few infected cells displayed a dendritic cell (DC) phenotype (CD11b^+^ Gr1^−^ F4/80^−^ CD11c^+^ and CD11b^−^ Gr1^−^ CD11c^+^ MHC class II^+^). The presence of macrophages and CD11b^+^ cells containing T. cruzi amastigotes in the cytoplasm was also confirmed histologically (Fig. 2b and c).

The apparently unchecked expansion of T. cruzi during the first ~8 days of infection (Fig. 1) suggested that monocyte/macrophages were acting primarily as a niche for parasite replication, rather than as effector cells limiting parasite growth. This conclusion was confirmed by the demonstration that depletion of macrophage populations by treatment with clodronate-containing liposomes resulted in a significant reduction in parasite numbers at the site of entry (Fig. 2d). In contrast, depletion of neutrophils by administration of 1A8 antibody did not modify the kinetics of the infection (Fig. 2e). Thus, T. cruzi appears to selectively invade and more productively replicate locally within macrophages/monocytes at the initial infection site.

### T. cruzi-infected host cells lack evidence of activation during the initial round of invasion and replication.

**FIG 3:**
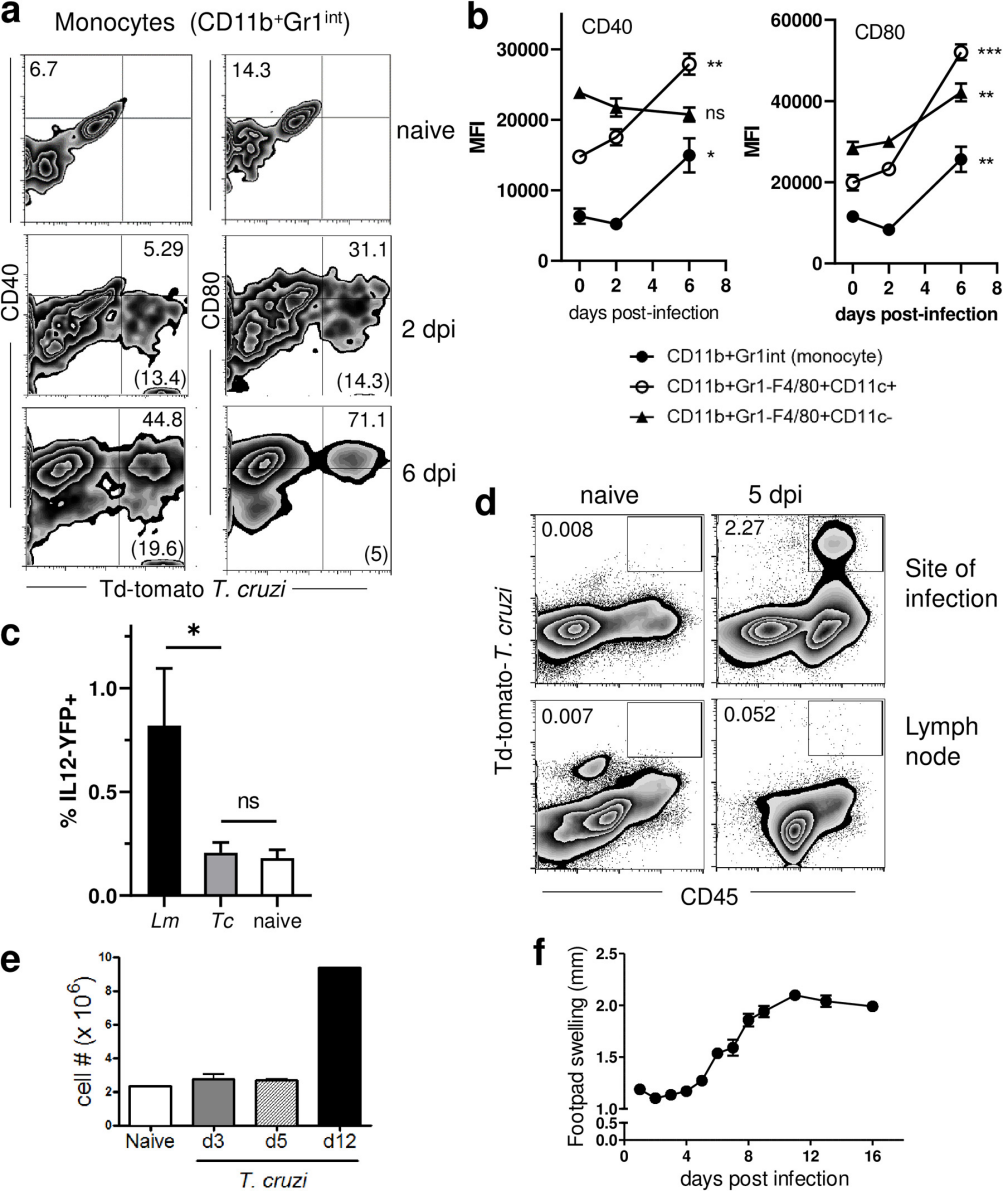
Lack of host cell activation during the first round of T. cruzi replication. (a and b) Host cells at the site of infection (ear) increased the levels of activation markers CD80 and CD40 only after 4 days postinfection, as detected by flow cytometry. Numbers in panel a represent the percentages of infected monocytes (numbers in parentheses) and the percentages of infected monocytes expressing the CD40 or CD80 activation markers (numbers at the top right). CD40 and CD80 expression on the surface of cells from the site was determined by flow cytometry at 2 and 4 days postinfection and compared to that of mock-injected animals (0 days postinfection) (b). Asterisks represent the statistical significance of the difference between 2 and 6 days postinfection. (c) IL-12-YFP reporter mice were infected in the ears with CL-tdTomato-expressing T. cruzi (*Tc*) or L. monocytogenes (*Lm*) (positive control), and the IL-12 expression by CD11b^+^ Gr1^−^ F4/80^+^ CD11c^+^ host cells at the site of infection and the draining lymph node was determined by flow cytometry at 2 days postinfection. (d) Cells containing CL-tdTomato-expressing T. cruzi were detected at the site of infection and the draining lymph node at 5 days postinfection by flow cytometry (numbers indicate the percentage of tdTomato^+^ cells of the total number of viable cells isolated). (e and f) Cell infiltration at the site of infection (e) and swelling of the infected footpads (f) also occurred after the first round of intracellular parasite replication. MFI, mean fluorescence intensity (geometric mean). *, *P* ≤ 0.05; **, *P* ≤ 0.01; ***, *P* ≤ 0.001; ns, not significant.

The early successful and apparently unrestricted expansion of T. cruzi within macrophages and monocytes at the infection site suggested that host cell invasion by T. cruzi failed to activate the potential antimicrobial and antigen-presenting capacity of these cells. This result is surprising, given that T. cruzi has been noted to be highly activating for macrophages *in vitro* (10, 11). In order to assess the activation status of these infected cells *ex vivo*, we first examined the expression of the activation markers CD40 and CD80 on T. cruzi-infected cells from the infection site. For all classes of potential APCs at the infection site (i.e., monocytes, macrophages, and DCs), expression of CD40 and CD80 on both T. cruzi-infected and noninfected cells was at the level of naive cells through 4 days postinfection and became significantly different by day 6 postinfection (Fig. 3a and b). Moreover, T. cruzi-infected CD11c^+^ cells from the infection site failed to express interleukin 12 (IL-12), as determined by the lack of yellow fluorescent protein (YFP)-expressing cells in IL-12-YFP reporter mice (Fig. 3c). Although cells containing tdTomato-expressing parasites were sometimes detected in the draining lymph nodes, the scarcity of these cells prevented us from rigorously assessing their activation status (Fig. 3d). Additional evidence of the absence of activating signals at the infection site prior to day 4 of infection comes from observation of the kinetics of infection site swelling and cellular infiltration. Again, both phenomena were absent during the first ~4 days of infection (Fig. 3e and f). At day 5 postinfection, the initial signs of an inflammatory response became apparent, with infiltration by several leukocyte populations, predominantly monocytes (Fig. 2a); by 6 days postinfection, both parasite-infected and noninfected host cells exhibited greater percentages of CD40/CD80^+^ cells and higher levels of expression of these molecules in the positive cells (Fig. 3a). These results firmly establish that, despite harboring rapidly replicating intracellular T. cruzi, potential antimicrobial and antigen-presenting cells at the site of T. cruzi infection are not activated during the initial round of cell invasion and replication. It is only after ~4 days of infection, a time point coincident with the end of the initial round of infection and the release of parasites (and the death of their former host cells), that cells of the innate immune system appear to be alerted to the presence of T. cruzi at the infection site.

### Parasite load at the site of entry is controlled by IFN-γ produced by infiltrating lymphocytes.

**FIG 4:**
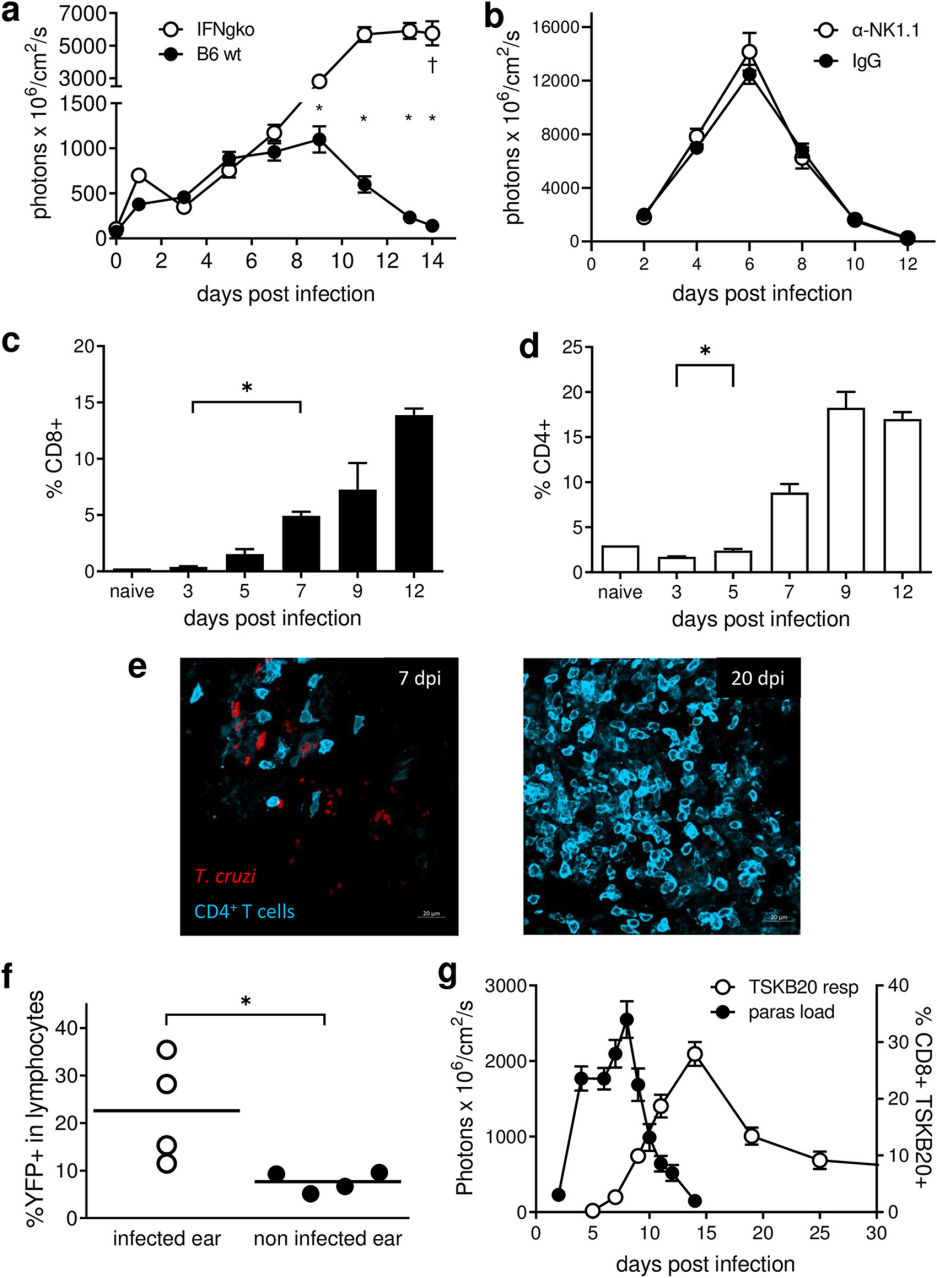
Parasite control at the site of entry coincides with the generation of an adaptive immune response. (a) IFN-γ-deficient (IFNgko) and C57BL/6 (B6 wt) mice were infected in the footpads with CL-tdTomato-expressing parasites, and the infection level at the site was measured by *in vivo* imaging until 14 days postinfection, when IFN-deficient mice had to be euthanized due to excessive pathology. (b) NK cell-depleting antibody against NK1.1 (PK136) and isotype control (IgG) were administered to C57BL/6 mice before and after the infection with tdTomato-expressing parasites in the footpads. (c and d) C57BL/6 mice were infected in the ears, and the proportions of CD8^+^ (c) and CD4^+^ (d) T cells infiltrating the site were determined by flow cytometry. Asterisks in panels c and d denote the time points at which the difference from the first sample became statistically significant (*P* < 0.05). (e) Maximum intensity projection images from whole mounted ears labeled with anti-CD4 antibody at 7 and 20 days after infection with tdTomato-expressing T. cruzi. (f) IFN-γ-YFP reporter mice were infected in one ear, and the percentages of YFP^+^ lymphocytes in the infected and contralateral ears were determined by flow cytometry. (g) Kinetics of parasite proliferation at the site of infection (paras load) and CD8^+^ T cell response specific to the T. cruzi peptide TSKB20 (TSKB20 resp) were tracked by *in vivo* imaging and MHC class I-TSKB20 tetramer complex staining of peripheral blood, respectively, in C57BL/6 mice infected in the footpads with CL-tdTomato-expressing parasites. *, *P* < 0.05.

The initial T. cruzi infection site shows evidence of activation by day 5 (Fig. 3), with a subsequent drop in parasite load >8 days postinfection (Fig. 1). The decrease in parasite level after that time point cannot be explained solely by parasite migration and colonization of distant tissues, since we detect parasite release starting as early as 5 days postinfection (see Video S4). More likely, this decrease is an outcome of activation and the initiation of effective immune control mechanisms. Evidence for this comes from the observation that mice deficient in IFN-γ fail to establish this early infection-site-specific control and develop uncontrolled parasite growth and pathology (Fig. 4a). Although natural killer (NK) cells are strong IFN-γ producers involved in the control of acute systemic T. cruzi infection (12), their depletion by administration of anti-NK1.1 antibody did not modify the kinetics of parasite load at the site of T. cruzi infection (Fig. 4b). The infiltration of the infection site by T cells (Fig. 4c to e) and the presence of IFN-γ-YFP^+^ lymphocytes in reporter mice coincide with the parasite control at the site (Fig. 4f), again supporting the hypothesis of active parasite control mediated by T cell-derived IFN-γ. Notably, the development of a parasite-specific CD8^+^ T cell response also coincides with the control of the parasite load at the site of primary infection (Fig. 4g).

**FIG 5:**
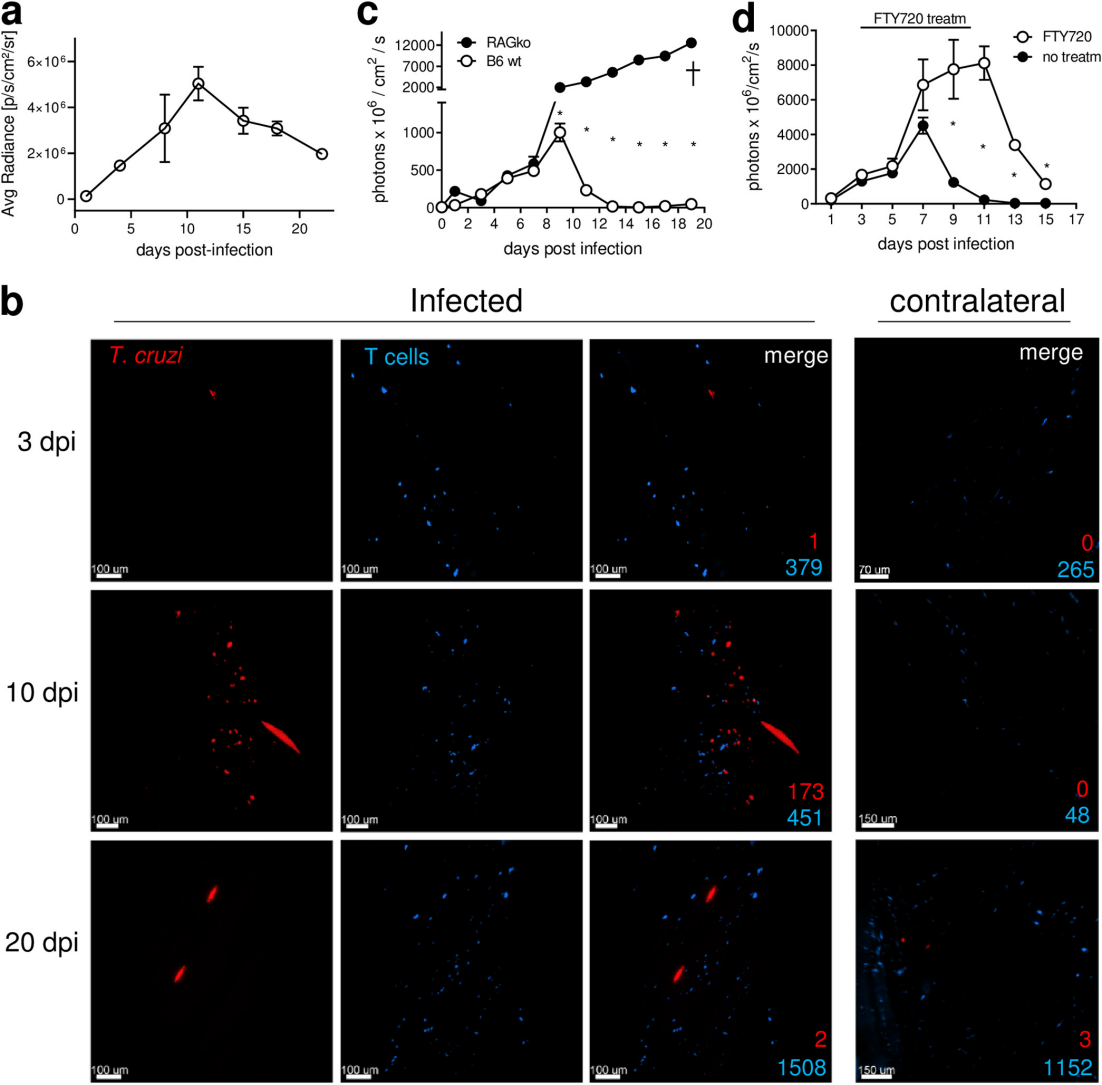
Parasites at the site of entry are controlled by infiltrating lymphocytes producing IFN-γ. (a) C57BL/6 mice were infected intramuscularly in the hind legs with 2.5 × 10^5^ trypomastigotes of Colombiana-T. cruzi expressing luciferase, and the parasite load at the site of infection was approximated by *in vivo* imaging of luciferase activity. Mice with fluorescently tagged T cells received an intramuscular injection of parasites expressing tdTomato and a mock phosphate-buffered saline (PBS) injection in the contralateral leg (contralateral). (b) Tissue samples from both legs were obtained early (day 3), near the peak of parasite load (day 10), and late (day 20) postinfection, clarified, and imaged by LSFM. The results of automated quantification of parasite-infected host cells (note that quantification of individual parasites within infected host cells is not possible at this magnification level [34]) and T cells in the muscle samples of infected or mock-injected muscle are indicated. (c) Lymphocyte-deficient (RAGko) and C57BL/6 (B6 wt) mice were infected in the footpads with CL-tdTomato-expressing parasites, and the infection level was measured by *in vivo* imaging. (d) C57BL/6 mice infected in the footpads with CL-tdTomato-expressing parasites were treated with FTY720 from day 2 to day 10 after infection, and the kinetics of parasite load at the site were measured by *in vivo* imaging. *, *P* < 0.05 (Mann-Whitney *U* test).

To more directly address the relationship between T cell infiltration and infection control, we developed a model of intramuscular infection (a preferred site of parasite replication in established T. cruzi infections) and followed T cell influx and parasite control *in situ* using light sheet fluorescence microscopy (LSFM). We first established, via detection of luciferase signal in these luciferase- and tdTomato-coexpressing parasites, that intramuscular infection resulted in similar kinetics of parasite proliferation and control, compared to those observed in the ear and footpad models; interestingly, however, the evolution of control and parasite clearance was substantially slower in muscle than in the ear and footpad, with a peak of parasite numbers at >10 days and detectable parasite load for >20 days (Fig. 5a). Imaging of clarified muscle tissue showed no increased accumulation of T cells at the infection site (relative to the contralateral uninfected leg) at day 3 postinfection, despite the presence of parasite-infected cells (Fig. 5b). In contrast, at 20 days postinfection, the number of T cells increased in concert with a reduction in parasite levels (Fig. 5a and b; also see Video S5 and Video S6). T cells also begin appearing in the control contralateral mock-infected leg by 20 days postinfection, with the spread of the systemic T. cruzi infection to this site (Fig. 5b).

10.1128/msphere.00601-22.3 Video S1 *In vivo* imaging of the ear of a hairless mouse 20 min after infection with tdTomato-expressing trypomastigotes. Download Movie S1, MOV file, 1.9 MB. Copyright © 2023 Padilla et al. 2023 Padilla et al. https://creativecommons.org/licenses/by/4.0/ This content is distributed under the terms of the Creative Commons Attribution 4.0 International license.

10.1128/msphere.00601-22.4 Video S2 *In vivo* imaging of the ear of a hairless mouse 24 h after infection with tdTomato-expressing trypomastigotes. Download Movie S2, MOV file, 0.7 MB. Copyright © 2023 Padilla et al. 2023 Padilla et al. https://creativecommons.org/licenses/by/4.0/ This content is distributed under the terms of the Creative Commons Attribution 4.0 International license.

10.1128/msphere.00601-22.5 Video S3 *In vivo* imaging of the ear of a hairless mouse 4 days after infection with tdTomato-expressing trypomastigotes. Download Movie S3, MOV file, 0.1 MB. Copyright © 2023 Padilla et al. 2023 Padilla et al. https://creativecommons.org/licenses/by/4.0/ This content is distributed under the terms of the Creative Commons Attribution 4.0 International license.

10.1128/msphere.00601-22.6 Video S4 *In vivo* imaging of the ear of a hairless mouse 5 days after infection with tdTomato-expressing trypomastigotes. Download Movie S4, MOV file, 9.2 MB. Copyright © 2023 Padilla et al. 2023 Padilla et al. https://creativecommons.org/licenses/by/4.0/ This content is distributed under the terms of the Creative Commons Attribution 4.0 International license.

10.1128/msphere.00601-22.7 Video S5 Three-dimensional reconstruction of LSFM images of skeletal muscle of mice at 10 days postinfection, showing tdTomato-expressing parasites (red) and ZsGreen-expressing cells (blue). Download Movie S5, MOV file, 0.5 MB. Copyright © 2023 Padilla et al. 2023 Padilla et al. https://creativecommons.org/licenses/by/4.0/ This content is distributed under the terms of the Creative Commons Attribution 4.0 International license.

10.1128/msphere.00601-22.8 Video S6 Three-dimensional reconstruction of LSFM images of skeletal muscle of mice at 20 days postinfection, showing tdTomato-expressing parasites (red) and ZsGreen-expressing cells (blue). Download Movie S6, MOV file, 0.7 MB. Copyright © 2023 Padilla et al. 2023 Padilla et al. https://creativecommons.org/licenses/by/4.0/ This content is distributed under the terms of the Creative Commons Attribution 4.0 International license.

Not surprisingly, mice deficient in lymphocytes failed to control parasite growth at the infection site (Fig. 5c), and retention of lymphocytes in the secondary lymphoid organs of infected C57BL/6 mice by S1P1 downregulation via FTY720 (fingolimod) treatment also interfered with the control of parasite load at the infection site (Fig. 5d). Together, these results establish that the infiltration of T cells in response to the local infection is key to controlling T. cruzi at the initial infection site, presumably through their production of IFN-γ.

### CD4^+^ T cells are the main source of IFN-γ for initial parasite control.

**FIG 6:**
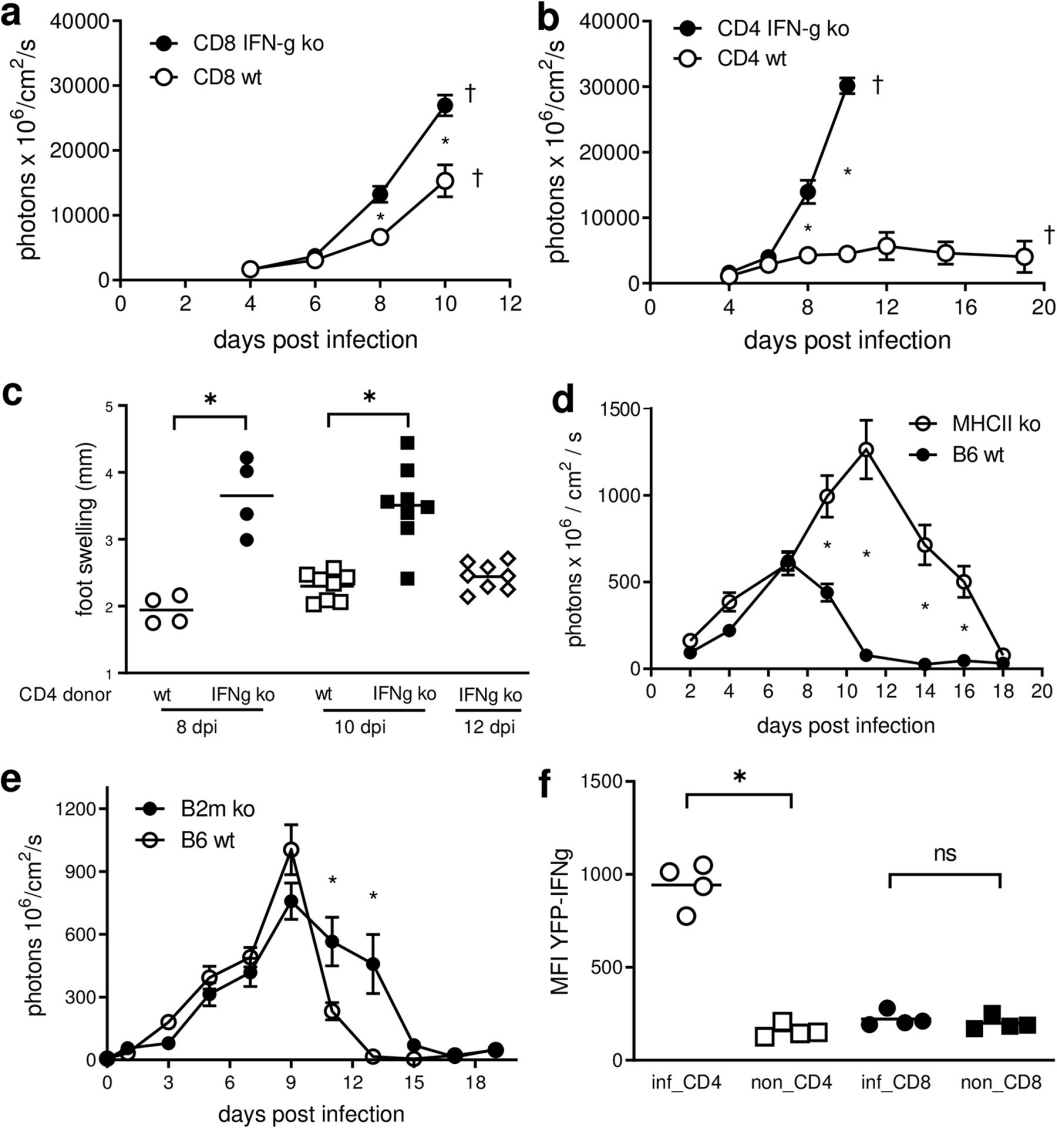
CD4^+^ T cells are the main source of IFN-γ for parasite control at the site of entry. (a and b) Sorted effector CD8^+^ (a) or CD4^+^ (b) T cells coming from IFN-γ-deficient (IFN-g ko) or C57BL/6 wild-type (wt) infected mice were transferred into IFN-γ-deficient mice the day prior to infection with CL-tdTomato-expressing parasites in the footpads, and parasite proliferation at the site was measured by *in vivo* imaging. (c) Local pathology in the animals of the groups receiving CD4^+^ T cells is expressed as swelling of the feet. (d and e) Mice deficient in CD4^+^ T cells (MHCII ko) (d), mice deficient in CD8^+^ T cells (B2m ko) (e), and C57BL/6 mice (B6 wt) were infected in the footpads with CL-tdTomato-expressing parasites, and the fluorescence at the site was measured by *in vivo* imaging. (f) Ear cells from the site of T. cruzi infection (9 days postinfection) of IFN-γ-YFP reporter mice were stained with antibodies against CD4 and CD8, and the mean fluorescence intensity (MFI) of YFP^+^ cells was measured by flow cytometry. *, *P* < 0.05; ns, not significant. †, time point at which the animals had to be euthanized due to excessive pathology.

To establish the specific source of IFN-γ responsible for the observed initial control of parasite load at the site of infection, we transferred sorted effector CD4^+^ or CD8^+^ T cells from T. cruzi-infected wild-type or IFN-γ knockout (KO) mice into IFN-γ-deficient C57BL/6 mice prior to infection with tdTomato-expressing T. cruzi. Although the transfer of wild-type effector CD8^+^ T cells resulted in a modest reduction of the parasite load at the site of infection, uncontrolled pathology necessitated termination of the experiment before 2 weeks postinfection (Fig. 6a). In contrast, primed wild-type CD4^+^ T cells provided substantial control of both parasites and pathology (Fig. 6b and c), doubling the survival time of the animals in this model. Likewise, mice deficient in CD4^+^ T cells exhibited less efficient control of parasite growth at the site of initial infection, relative to mice deficient in CD8^+^ T cells (Fig. 6d and e), although, as reported previously (13, 14), both CD4^+^ and CD8^+^ T cell-deficient mice ultimately failed to control the resulting systemic infection. CD4^+^ T cells at the site of initial parasite infection in IFN-γ-YFP reporter mice displayed a higher mean fluorescent intensity than CD8^+^ T cells at the site (Fig. 6f), indicative of cells actively producing IFN-γ (15). Thus, CD4^+^ T cells appear to be the principal IFN-γ-producing cells at the initial infection site and are crucial to the control of T. cruzi at the site of entry. Notably, however, neither the transfer of parasite-primed CD4^+^ or CD8^+^ T cells (Fig. 6a and b) nor the total absence of CD4^+^ or CD8^+^ T cells (Fig. 6d and e) impacted parasite load at the site of infection during the initial 6 to 7 days of infection.

### Previously primed memory T cells are also slow to reactivate after challenge infection.

**FIG 7:**
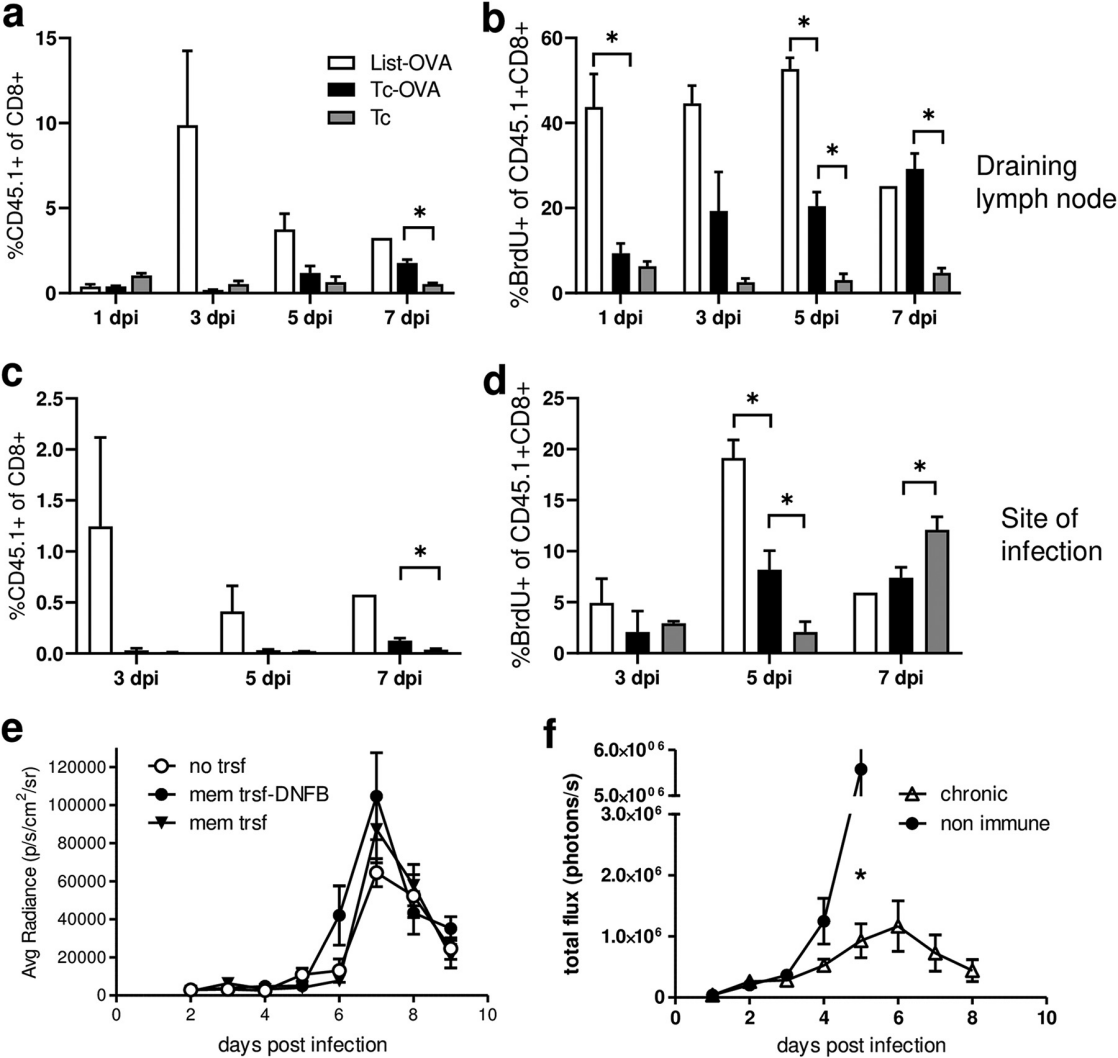
Previously primed T memory cells are also slow to reactivate after challenge infection. CD45^+^ OT-I cells were transferred to naive CD45.2 C57BL/6 mice prior to activation by intravenous injection of OVA-expressing Listeria monocytogenes. Four weeks after OT-I cell activation, mice were infected in the ears with wild-type Brazil-T. cruzi (Tc), OVA-expressing Brazil-T. cruzi (Tc-OVA), or OVA-expressing Listeria monocytogenes (List-OVA). (a to d) Bromodeoxyuridine (BrdU) was injected 24 h before collection of draining lymph node (a and b) and site of infection (c and d) samples. Cell suspensions were stained with fluorescent antibodies against CD45.1 to identify the OT-I population and with anti-BrdU to determine proliferation (b and d). (e) C57BL/6 mice that had previously received draining lymph node cells from acutely infected animals without treatment (mem trsf) or with DNFB treatment in the ears (mem trsf-DNFB) were infected with luciferase-expressing parasites, and the parasite loads over time were compared to those of mice that did not receive cells (no trsf). (f) Chronically infected (chronic) or naive (non immune) C57BL/6 mice were infected in the footpads with luciferase-expressing T. cruzi, and the parasite loads at the site of infection were measured over time by *in vivo* imaging. The asterisk in panel f denotes the first time point at which the luminescence intensities of the chronic and nonimmune groups were statistically different. *, *P* < 0.05.

Although the T cell response at the site of infection is relatively slow to develop, it is nonetheless effective in ultimately controlling the infection at that site, although not before the infection has spread to other host tissues. An effective vaccine designed to prime antipathogen T cells would require that memory T cells systemically, or locally at the infection site if they are present, respond to and control pathogens soon after infection, before they spread to other sites. To determine whether previously primed memory T cells are rapidly responsive to T. cruzi infection at the infection site, irrespective of host cell types infected there, we generated ovalbumin (OVA)-specific OT-I central memory T cells in wild-type mice and then infected these mice with either T. cruzi-OVA or *Listeria*-OVA (control). In mice infected with *Listeria*-OVA, reactivation of the CD45.1 congenically marked OT-I memory population was evident as early as 3 days postinfection, as demonstrated by a rapid increase in the proportion of these cells in the draining lymph node (Fig. 7a) and at the site of infection (Fig. 7c). In contrast, expansion of OT-I cells in the lymph node and at the site of infection in memory mice infected with T. cruzi-OVA was not significantly increased until ~5 to 7 days postinfection (Fig. 7a and c). The relatively delayed activation of OT-I cells in the T. cruzi-OVA-infected animals was also confirmed by the reduced proliferation, compared to OT-I cells in animals infected with *Listeria*-OVA (Fig. 7b and d).

To further enhance the potential of T. cruzi-specific memory cells specifically at the site prior to parasite infection, we first transferred early activated cells from the draining lymph nodes of T. cruzi-infected mice into naive mice and then induced the mobilization of these cells to the future infection site using 1-fluoro-2,4-dinitrobenzene (DNFB) treatment (16). Mice infected with tdTomato-expressing T. cruzi at the site of T cell recruitment showed no enhancement of control of the parasites at this site, relative to mice that did not receive T cells or that received transferred T cells but not DNFB (Fig. 7e). Finally, mice with an ongoing but well-controlled T. cruzi infection, and thus with potent effector T cell responses (17), exhibited no enhanced parasite control during the initial 4 to 5 days after the introduction of a new infection in the skin, compared to naive mice (Fig. 7f). In these chronically infected animals, the new infection was effectively controlled but only after day 4, corresponding to the end of the first round of intracellular replication and destruction of the host cells. These results indicate that, even in the presence of a specific and highly potent, infection-controlling immune response against T. cruzi, newly infecting parasites can readily establish in the “immune” host.

## DISCUSSION

Recognizing and marshalling rapid responses to pathogens soon after host entry are crucial for both the speedy development of immunity upon the initial encounter with a pathogen and protection from infection in sterilely immune or vaccinated hosts. In the case of T. cruzi, these initial encounters occur at the site of parasite entry, which in naturally acquired infection is often the mucosa or breaks in the skin. Previous studies reported a relative lack of response to T. cruzi soon after infection in the skin, with no macroscopic evidence of inflammation up to 7 days postinfection and a modest influx of CD11b^+^ leukocytes (18–20), although this pattern may be modified by the presence of insect feces or the infective parasite form (21–23). The response to T. cruzi gains momentum and ultimately constrains the infection, but this process is slower than might be expected (3, 24) and, perhaps as a result, the infection rapidly disseminates systemically, where it is persistent (25, 26).

In this study, we have further investigated these early interactions after infection in the skin, monitoring both parasite development and host immune responses at the infection site and systemically. A conclusive result from this investigation is that the initial phase of *in vivo* host cell invasion and replication by T. cruzi occurs with minimal recognition by either the infected host cells or the immune system overall. Monocytes/macrophages, both resident at the infection site and recruited to the site as the infection progresses, are the predominant cells hosting T. cruzi at these early points but exhibit few signs of activation. Rather, these potential drivers and mediators of parasite control appear to be important for infection success, as their depletion significantly reduces parasite numbers at the infection site.

Although immune control of T. cruzi eventually comes to the infection site, this occurs well after parasites have distributed systemically into tissues where they are known to persist, usually for the life of the host (25, 26). The infection site control is, as expected, IFN-γ dependent and is primarily mediated by CD4^+^ T cells, the latter perhaps reflective of the fact that most of the infected cells at the site are capable of expressing class II MHC. In contrast, control of the infection systemically and in the long term requires a strong contribution of CD8^+^ T cells (14) and their apparent recognition of host cells such as muscle, which are negative for class II MHC. Although muscle may only rarely be a natural site of initial infection, the much slower time course of parasite expansion and control there, in comparison to skin, is interesting and may also be reflective of the long-term success of T. cruzi in this tissue. Why muscle is the major site of T. cruzi persistence is not known, but one contributor to parasite success at this site is the relatively low level of class I MHC in muscle (27).

One might expect that many hosts could recover from a delayed initial response to infection by ultimately mounting a robust systemic response, and this is indeed the case in T. cruzi infection. However, despite the strength of this anti-T. cruzi response, it is rarely sterilizing. We think that the sluggish response at “new” infection sites may be repeated constantly throughout T. cruzi infection. Thus, even when primed responder cells are present at the infection site, recognition that parasites have arrived at that site takes at least one round of host cell infection and parasite expansion, thus providing the opportunity for establishment of the infection and its ultimate dissemination. This could mean that each invasion of an immunologically quiescent site, even in an immune host, is followed by one or more rounds of parasite replication before immune effectors quell that expansion, although not before some parasites have moved on to other virgin sites. This scenario is also supported by the ability to establish new infections in previously cured or persistently infected mice, including those with multiple rounds to infection and cure (28). This pattern is a clear prescription for persistence.

If T. cruzi can continuously establish new infection sites in an infected host, despite a strong and controlling immune response (17, 28), then the chances seem rather low for a vaccine to routinely prevent infection. At best, vaccination may reduce the severity of an acute infection or perhaps reduce the infection success rate in cases in which very low numbers of parasites are introduced, but consistently preventing new infections in general may be too much to expect from even the best vaccine.

## MATERIALS AND METHODS

### Mice, parasites, and infections.

Mice deficient in CD8^+^ T cells (B6.129P2-B2mtm1Unc/J), IFN-γ reporter mice (B6.129S4-Ifngtm3.1Lky/J [IFN-γ eYFP “GREAT”]), B6.IL-12yet40 reporter mice (IL-12yet40) and IFN-γ-deficient mice (B6.129S7-Ifngtm1Ts/J) were purchased from the Jackson Laboratory (Bar Harbor, ME). C57BL/6NCr mice were purchased from Charles River Laboratories. SKH-1 “hairless” mice backcrossed to C57BL/6 were a gift from Lisa DeLouise (University of Rochester). Lymphocyte-deficient mice (RAGko), class II MHC-deficient mice (B6.129-H2-Ab1) and wild-type B6129F1/Tac mice were purchased from Taconic (Germantown, NY). RAG^−/−^ OT-I TCR (CD45.1) transgenic mice were obtained from Kimberly Klonowsky (University of Georgia). Mice with the Cd8a gene driving the expression of Cre recombinase (Cd8a-Cre) and Cre reporter mice (Ai6, RCL-ZsGreen) with a *loxP*-flanked STOP cassette preventing transcription of an enhanced green fluorescent protein variant (ZsGreen1) were purchased from the Jackson Laboratory and crossed in our facility to produce mice fluorescently tagged in all of the cells that express CD8a at any point of their development (including CD4^+^ and CD8^+^ T lymphocytes). Male and female animals (3 to 6 per group) were used in the experiments. All mice were maintained in the University of Georgia animal facility under specific-pathogen-free conditions. This study was carried out in strict accordance with the Public Health Service Policy on Humane Care and Use of Laboratory Animals and Association for Assessment and Accreditation of Laboratory Animal Care accreditation guidelines. The protocol was approved by the University of Georgia Institutional Animal Care and Use Committee.

T. cruzi trypomastigotes of the CL or Colombiana strain expressing the fluorescent protein tdTomato or the luciferase enzyme (8), parasites of the Brazil strain expressing OVA (29), and wild-type parasites of the TCC strain (30) were maintained in tissue culture by serial passage through Vero cells and were used for subcutaneous infection in the mouse ears or hind leg footpads and for intramuscular infections in the gastrocnemius muscle. Listeria monocytogenes expressing OVA (31) was obtained from K. Klonowski (University of Georgia, Athens, GA), and 10^3^ bacteria per mouse were intravenously injected. Footpad thickness was measured with a digital caliper at different times postinfection and related to preinfection measurements of the same footpad to determine the swelling.

### *In vivo* imaging.

Luciferase- and tdTomato-expressing parasites were used to assess the *in vivo* kinetics of parasite replication and control. Mice were subcutaneously infected in the ears or footpads with 2.5 × 10^5^ tdTomato-expressing trypomastigotes, in a total volume of 2 μL per ear or 4 μL per footpad. Fluorescent tdTomato signal at the site of infection was measured with a Maestro 2 *in vivo* system (CRi, Woburn, MA) using a green set of filters, as described previously (32). *In vivo* videos of tdTomato-expressing parasites in the ears of anesthetized hairless mice were obtained using an Olympus OV100 imaging system. For bioluminescence detection, mice were injected intraperitoneally with d-luciferin (150 mg/kg; PerkinElmer, Waltham, MA) and anesthetized using 2.5% (vol/vol) isofluorane in oxygen prior to imaging with an IVIS Lumina II imager (Xenogen, Alameda, CA), as described previously (8). Quantification of bioluminescence and data analysis were performed using Living Image v4.3 software (Xenogen).

### rtPCR.

Parasite equivalents at the site of infection (footpad) and in skeletal muscle, heart, and fat tissue were determined by rtPCR at different times postinfection, as described previously (33). Briefly, tissue samples were finely minced and incubated at 55°C in SDS-proteinase K lysis buffer. DNA was extracted twice with phenol-chloroform-isoamyl alcohol (25:24:1, by volume), precipitated with 100% ethanol, and resuspended in nuclease-free water. PCRs containing iQ SYBR green Supermix (Bio-Rad, Irvine, CA) and primers specific for T. cruzi or mouse genomic DNA were analyzed on an iCycler (Bio-Rad), and T. cruzi equivalents were calculated as the ratio of T. cruzi satellite DNA to the quantity of mouse tumor necrosis factor alpha (TNF-α) DNA in each sample.

### Cell isolation and phenotyping.

For isolation and phenotyping of cells at the site of infection, dorsal and ventral surfaces of infected ears were split using fine tweezers, and the exposed internal surface was placed for 20 min at 37°C on top of Hanks balanced salt solution (HBSS) with Ca^2+^ and Mg^2+^ containing 0.4 U/mL TL Liberase (Sigma-Aldrich, St. Louis, MO) and 80 U/mL DNase, on a shaker. Digestion was stopped by the addition of 1 mM EDTA, and the cell suspension was passed through a 70-μm nylon cell strainer (BD Biosciences, San Jose, CA) and pelleted via centrifugation. The cell suspension was labeled with specific antibodies and live/dead Aqua viability stain (Thermo Fisher Scientific, Waltham, MA). Cell numbers were determined by microscopic counting with a hemocytometer, and samples were analyzed by flow cytometry without fixation. Isolated cells were labeled with different antibody panels combining CD45-fluorescein isothiocyanate (FITC), CD11b-allophycocyanin-Cy7, Gr1-peridinin chlorophyll protein-cyanine 5.5 (PerCP5.5), CD11c-allophycocyanin, CD40-FITC, CD80-FITC, F4/8-phycoerythrin (PE)-Cy7, and MHC class II (I-A/I-E)-Pacific Blue (BioLegend, San Diego, CA). Cell suspensions from digested tissues were gated into live cells (see Fig. S1 in the supplemental material), single cells, CD45^+^ cells, and cells containing parasites (tdTomato^+^) or not. CD8^+^ T cells specific for T. cruzi from peripheral blood were determined by staining with the MHC class I tetramer containing the TSKB20 peptide (ANYKFTLV/Kb), which was synthesized at the Tetramer Core Facility (Emory University, Atlanta, GA), and anti-CD8-ef450 antibody. Antibodies against CD4, CD11b, and B220 labeled with PE-Cy5 (BioLegend) were used for lineage exclusion staining. Cell samples were collected on a CyAn ADP cytometer using Summit v4.3 (Beckman Coulter), and the data were analyzed with FlowJo flow cytometry software (Tree Star, Ashland, OR).

10.1128/msphere.00601-22.1 FIG S1 Gating strategy for flow cytometry analysis. Download FIG S1, PDF file, 0.7 MB. Copyright © 2023 Padilla et al. 2023 Padilla et al. https://creativecommons.org/licenses/by/4.0/ This content is distributed under the terms of the Creative Commons Attribution 4.0 International license.

### Microscopy.

Mice were infected with tdTomato-expressing parasites in the ears, and samples were collected for light or fluorescence microscopy 3 to 4 days postinfection. For light microscopy, samples were fixed in paraformaldehyde (4% [vol/vol]), sectioned with a microtome, and stained with hematoxylin and eosin for microscopic analysis. For fluorescence microscopy, ear samples were digested and labeled with fluorescent antibodies as described previously for the cell isolation process, and the labeled cells were collected by centrifugation and placed on a slide with a coverslip for fluorescence microscopy analysis with a Delta Vision microscope system II (GE Healthcare, Feasterville-Trevose, PA). LSFM of infected muscle was performed with clarified samples, which were processed, imaged, and quantified as described by Bustamante et al. (34). For confocal microscopy, whole ears were processed as described previously (35), labeled with anti-CD4 clone RM4-5 primary antibody and Alexa Fluor 647-labeled anti-rat IgG secondary antibody (BioLegend), mounted on slides with ProLong Diamond antifade mountant (Thermo Fisher Scientific), and imaged using a Zeiss LSM 710 confocal microscope. Confocal z-stack images ranging from 11 to 15 μm were processed with Zen 2011 software (Zeiss) to obtain the maximum intensity projections.

### Cell depletion and treatments.

C57BL/6 mice were injected in the footpads with 10 μL of clodronate-containing liposomes (Encapsula NanoSciences, Brentwood, TN) at −1, 3, 5, 7, and 9 days postinfection, with 2.5 × 10^5^ tdTomato-expressing trypomastigotes in each footpad. At 1 day postinfection, mice were also injected intravenously with 100 μL of clodronate-containing liposomes. Control mice were inoculated in a similar schedule with empty liposomes (see Fig. S2a). Neutrophil-depleting antibody (1A8) (see Fig. S2b), NK1.1-depleting antibody (PK136) (see Fig. S2c), and isotype control (IgG) were intravenously administered to C57BL/6 mice before and after the infection with tdTomato-expressing parasites in the footpads. C57BL/6 mice were treated by the oral route with 1 mg/kg FTY720, dissolved in distilled water (36), daily from day 2 to day 10 after infection with tdTomato-expressing trypomastigotes in each footpad. Fluorescence levels in the footpads were measured every other day, and the level of lymphocytes in blood was determined by flow cytometry at 10 days postinfection. For DNFB (Sigma-Aldrich) sensitization 5 days before cell transfer, mouse abdomens were shaved and depilated before the application of 25 μL of 0.5% DNFB in acetone-oil (4:1 [vol/vol]) to a 1-cm^2^ area of skin (16). Three days following cell transfer and L. monocytogenes injection, the ears of the sensitized animals were treated with 15 μL of 0.5% DNFB in acetone for activated cell recruitment; 4 weeks later, mice were infected with T. cruzi in the ears.

10.1128/msphere.00601-22.2 FIG S2 Cell depletion. (a) Infected C57BL/6 mice were treated with clodronate-containing or empty liposomes (negative control) as described in Materials and Methods. The depletion of the CD11b^+^ cell population and the CD11c^+^ and F4/80^+^ resident macrophage populations (CD11b^+^ Gr1^−^ F4/80^+^) was determined at 4 days postinfection by flow cytometry. (b and c) C57BL/6 mice were treated with 1A8 (b) or PK136 (c) antibodies as described in Materials and Methods, and the depletion of the circulating neutrophil and NK cell populations, respectively, was determined by flow cytometry in the peripheral blood at different times postinjection. Download FIG S2, PDF file, 0.5 MB. Copyright © 2023 Padilla et al. 2023 Padilla et al. https://creativecommons.org/licenses/by/4.0/ This content is distributed under the terms of the Creative Commons Attribution 4.0 International license.

### Adoptive cell transfers.

OT-I cells (CD45.1) were transferred to naive CD45.2 C57BL/6 mice 24 h prior to activation by intravenous injection of OVA-expressing Listeria monocytogenes. Mice were infected with T. cruzi trypomastigotes in the ears 4 weeks after OT-I cell transfer and OVA-expressing L. monocytogenes injection. For transfer of early activated cells, C57BL/6 mice were infected with T. cruzi in the ears, and cells from the auricular draining lymph nodes were collected at 10 days postinfection and transferred intravenously into DNFB-sensitized mice. DNFB (Sigma-Aldrich) treatment in the ears of recipient mice was performed 5 days prior and 1 day after cell transfer, and mice were infected in the ears with luciferase-expressing T. cruzi 4 weeks after cell transfer. To avoid any possible transfer of parasites along with the draining lymph node cells, recipient mice received two oral doses of benznidazole (100 mg/kg; Elea Phoenix) on days −2 and 0 of the cell transfer. For transfer of lymphocyte subpopulations, spleens from C57BL/6 and IFN-γ KO mice chronically infected with CL and TCC T. cruzi strains, respectively, were mechanically homogenized with frosted glass slides in a hypotonic ammonium chloride buffer for lysis of the red blood cells and were washed in RPMI 1640 medium with 10% fetal bovine serum (FBS). CD8^+^ and CD4^+^ T cells were sorted by fluorescence-activated cell sorting (FACS) with a MoFlo sorter (Beckman Coulter). Sorted cells (10^6^) of the different populations were transferred intravenously into IFN-γ KO mice 24 h prior to infection with tdTomato-expressing T. cruzi in the footpads.

### Statistical analysis.

The Mann-Whitney and unpaired *t* tests of GraphPad Prism v5.0 were used. Values are expressed as means ± standard errors of mean. *P* values of ≤0.05 were considered significant.
