# Supplementary material for: Delayed Activation of T Cells at the Site of Infection Facilitates the Establishment of Trypanosoma cruzi in Both Naive and Immune Hosts
Source: mSphere. 2023 Jan 25;8(1):e00601-22. doi: 10.1128/msphere.00601-22 (PMC9942555; doi:10.1128/msphere.00601-22)

S1 Fig. Gating strategy for dissecting different cell populations in the footpad.

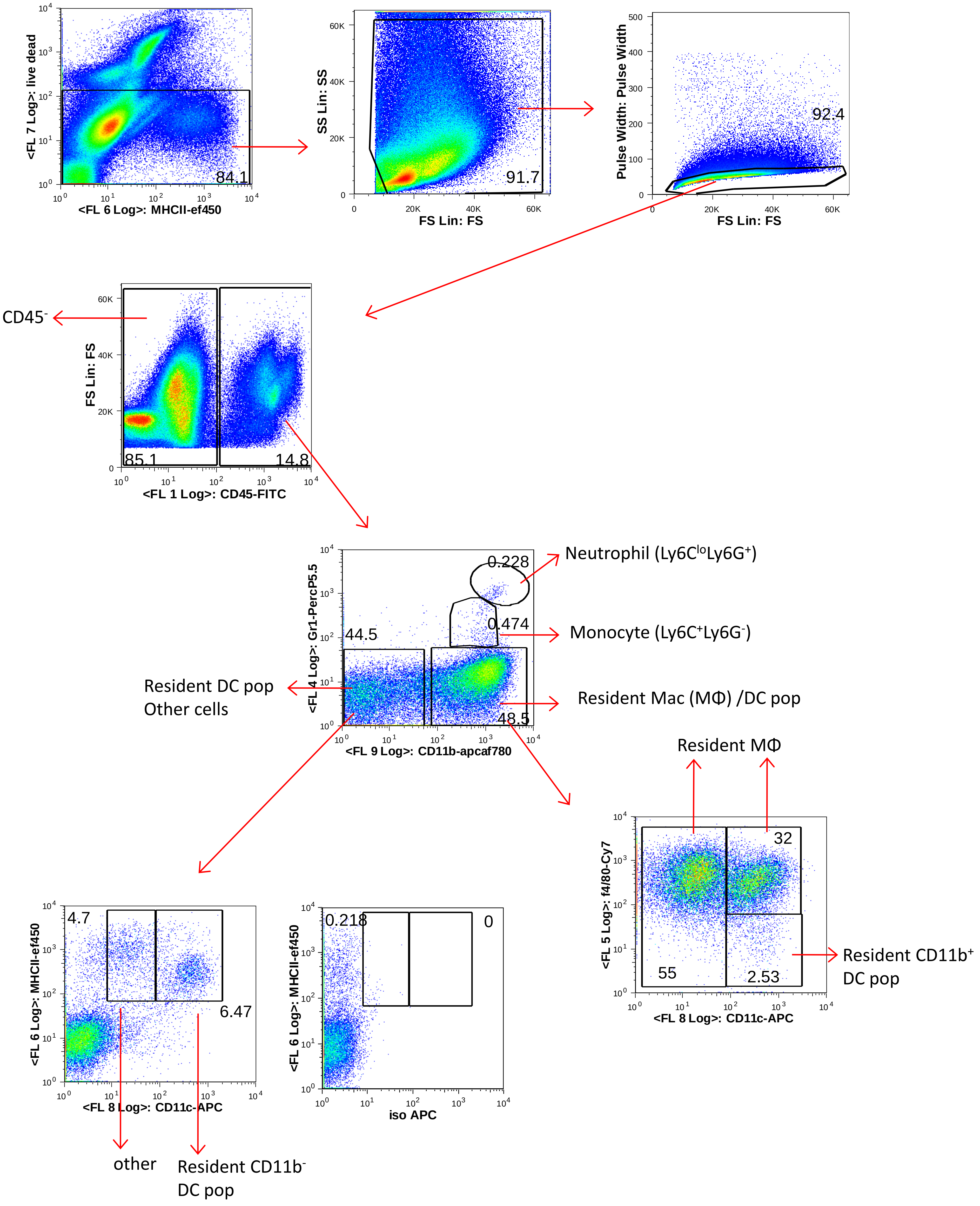

<FL 4 Log>

Gating strategy for identifying IFNg-YFP+ cells at the site of infection

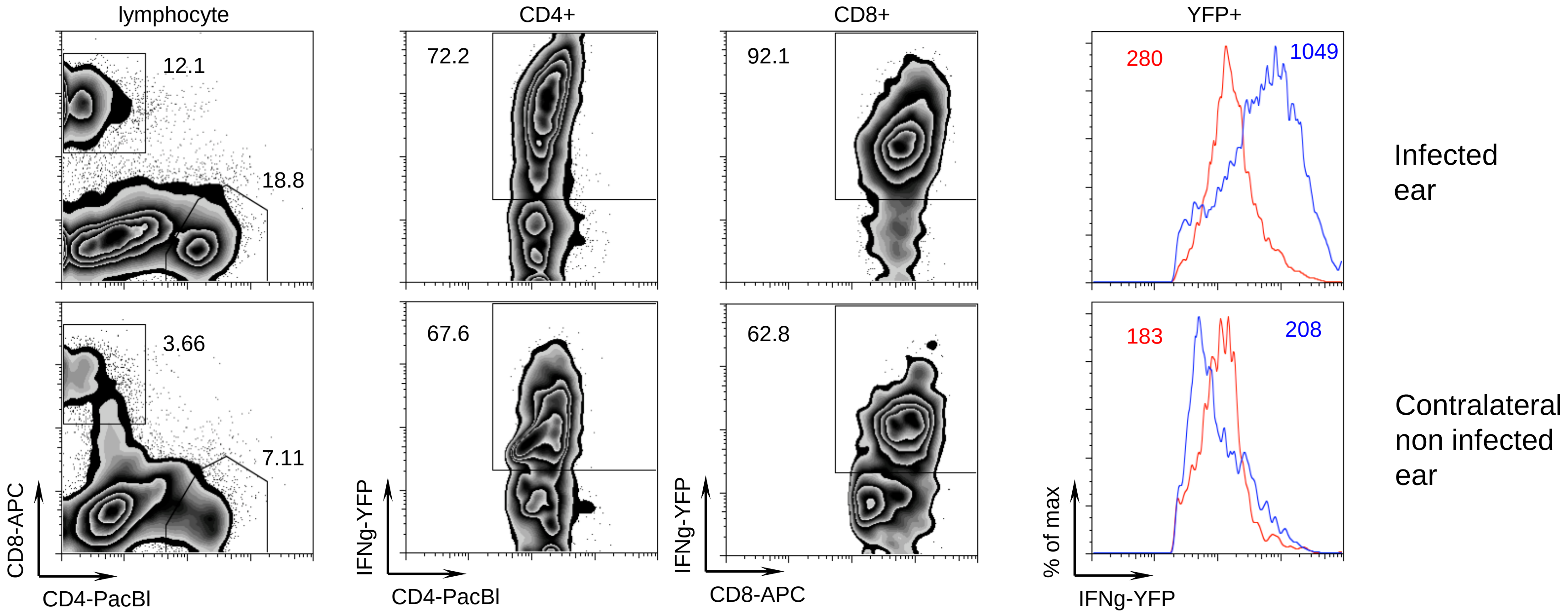

Gating strategy for identifying TSKB20 specific CD8+ T cells in peripheral blood

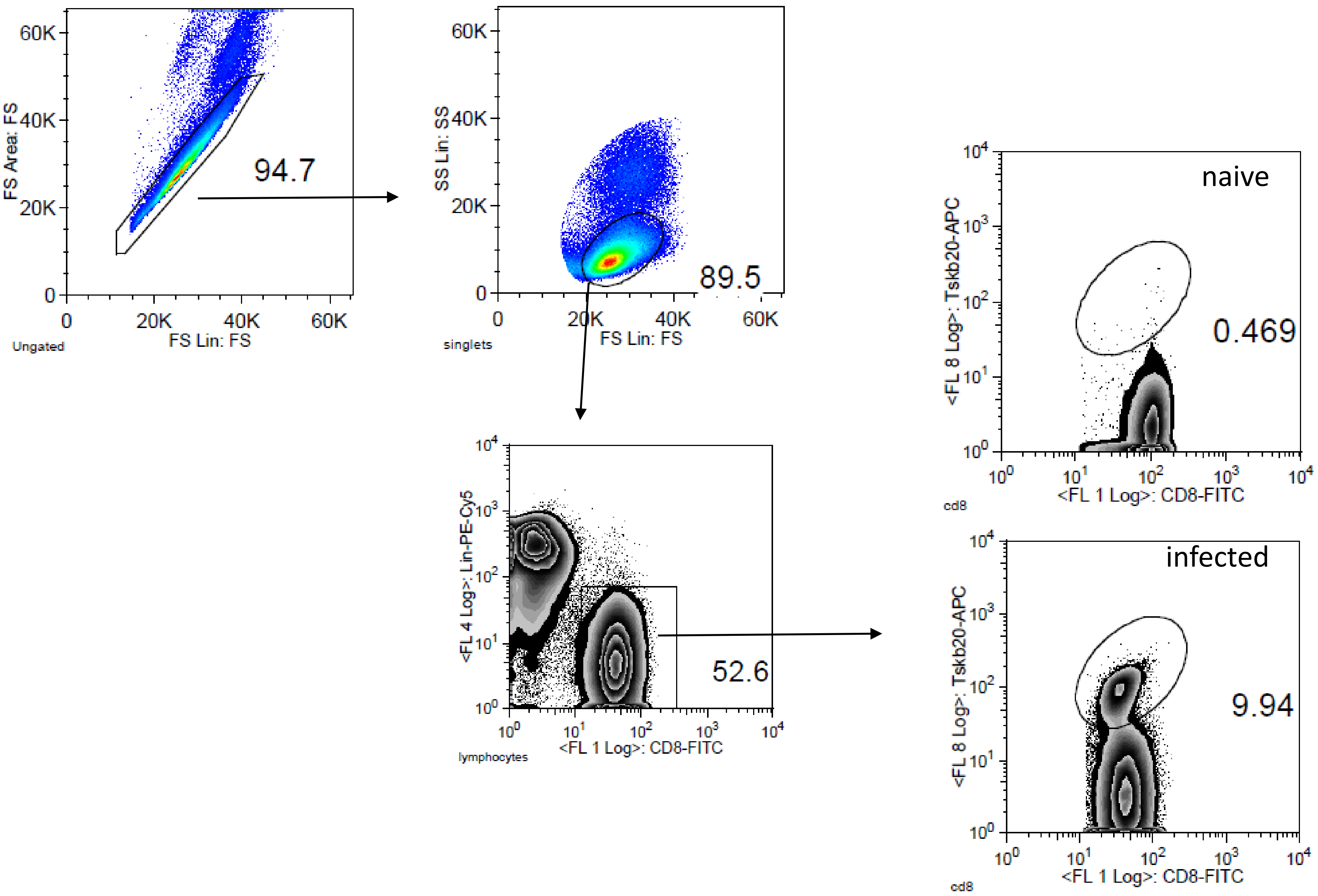

Supplement: FIG S1 [file msphere.00601-22-s0002.pdf]
