# Supplementary material for: Delayed Activation of T Cells at the Site of Infection Facilitates the Establishment of Trypanosoma cruzi in Both Naive and Immune Hosts
Source: mSphere. 2023 Jan 25;8(1):e00601-22. doi: 10.1128/msphere.00601-22 (PMC9942555; doi:10.1128/msphere.00601-22)
Supplement: FIG S2 [file msphere.00601-22-s0001.pdf]

S2_Fig. Confirmation of depletion of cellular populations

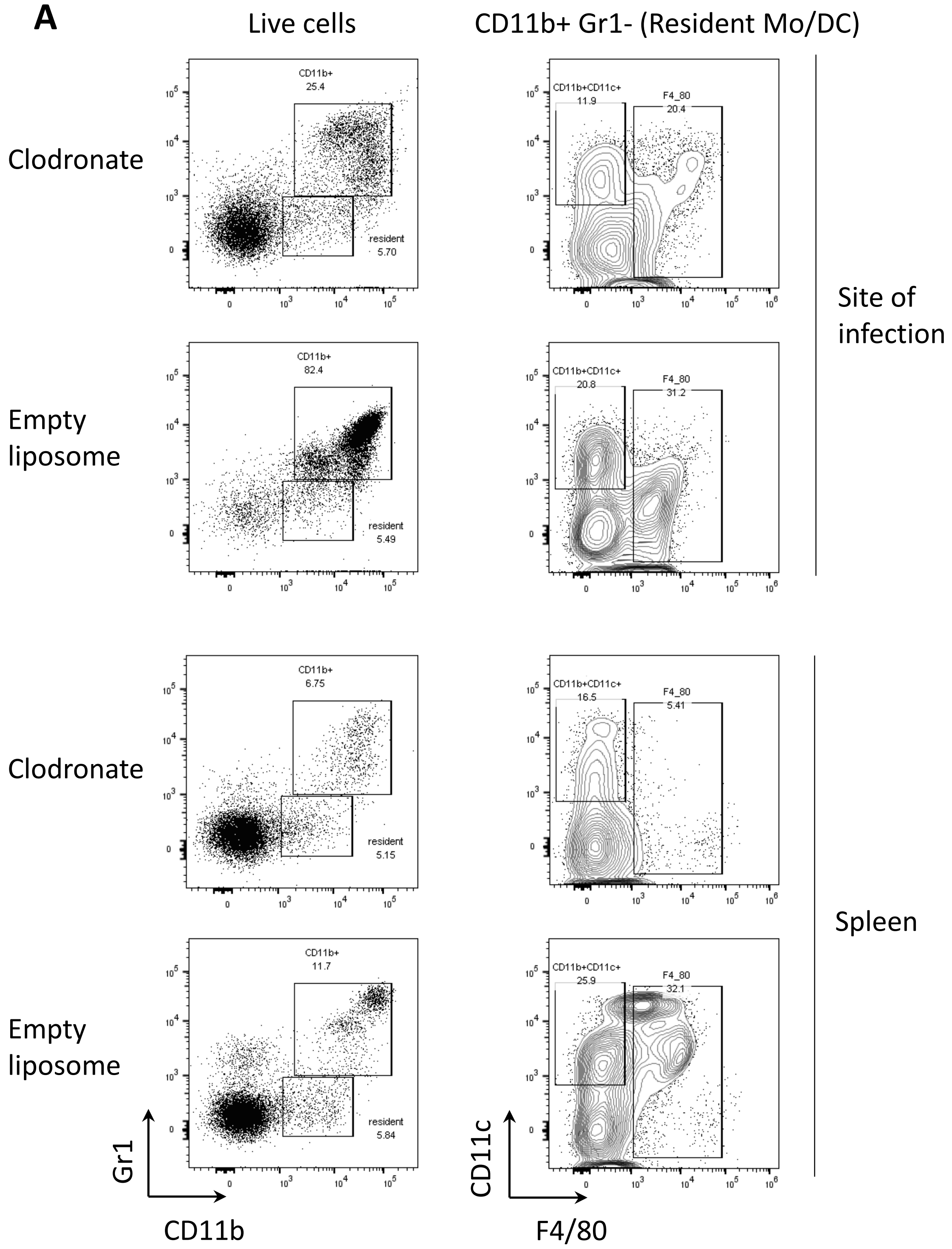

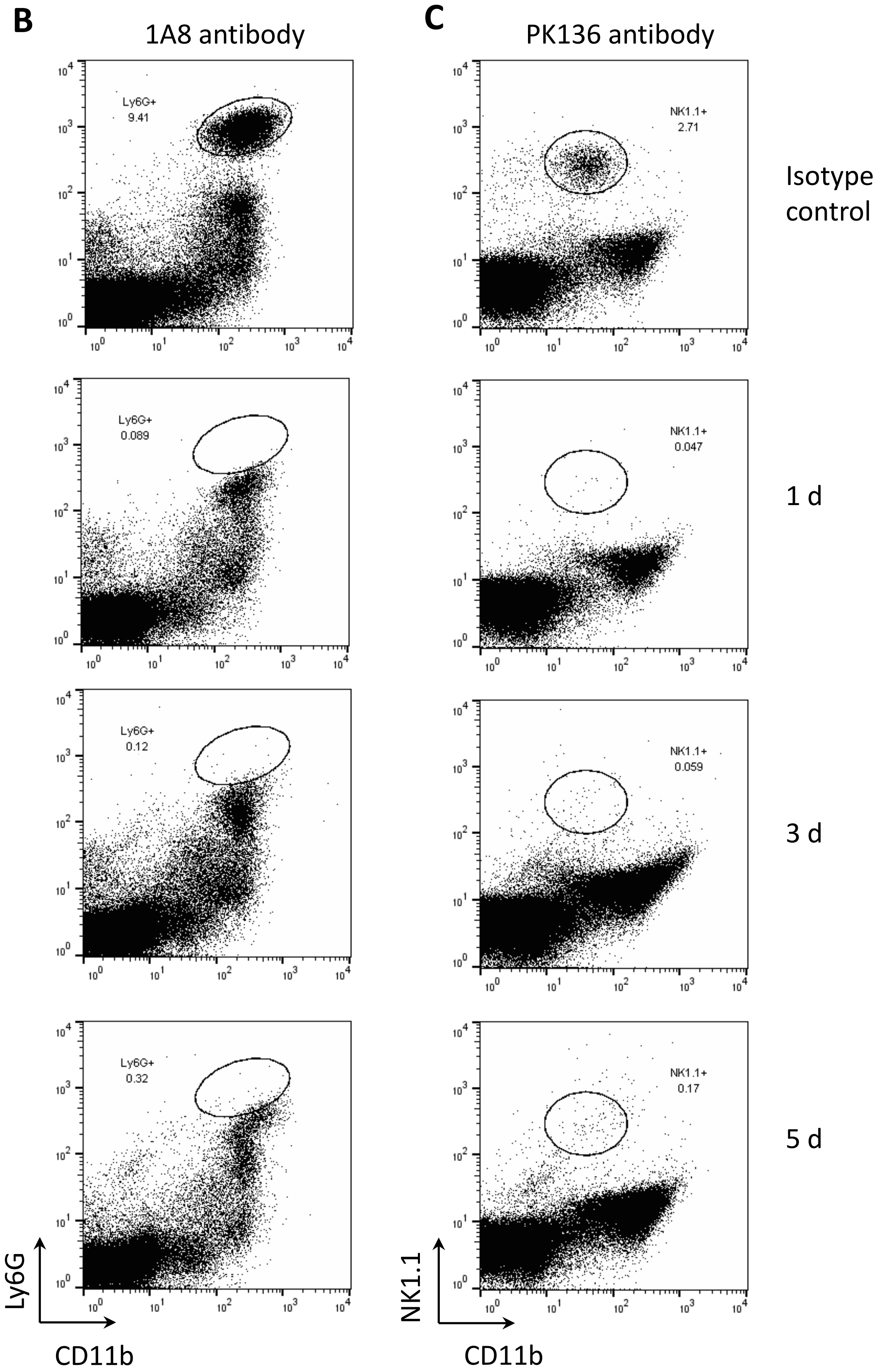

B
1A8 antibody
C
PK136 antibody
Ly6G+
9.41
NK1.1+
2.71
Isotype control
Ly6G+
0.089
NK1.1+
0.047
1 d
Ly6G+
0.12
NK1.1+
0.059
3 d
Ly6G+
0.32
NK1.1+
0.17
5 d
Ly6G
CD11b
NK1.1
CD11b
